# Uncovering the Hidden Threat: A Case Study on Superior Vena Cava Thrombosis

**DOI:** 10.7759/cureus.37040

**Published:** 2023-04-02

**Authors:** Nabeel Karim, Gaelle Laurore-Fray

**Affiliations:** 1 Dr. Kiran C Patel College of Osteopathic Medicine, Nova Southeastern University, Clearwater, USA; 2 Graduate Medical Education Director/Family Medicine, Tampa Family Health Centers, Tampa, USA

**Keywords:** undiagnosed, rare, high-risk pregnancy, transient ischemic attack (tia), superior vena cava thrombus

## Abstract

Superior vena cava (SVC) thrombus is a rare but potentially life-threatening condition where a blood clot forms in the superior vena cava, the vein carrying blood from the head, neck, and upper extremities to the heart. The incidence of SVC thrombosis is higher in patients with certain underlying medical conditions, such as malignancy, heart failure, and chronic obstructive pulmonary disease. In this case study, a 36-year-old African American female with a history of essential hypertension, type 2 diabetes, end-stage renal disease, anemia of chronic disease, obstructive sleep apnea, obesity, and preeclampsia presented with sudden onset of confusion six days postpartum. The patient was admitted for further evaluation and treatment. Imaging tests showed an acute infarct in the left parietal lobe with no intracranial hemorrhage and an echo density/mass in the SVC consistent with a thrombus. Risk factors for SVC thrombus included pregnancy, a hypercoagulable state, and issues with catheter placement. The increasing use of intravascular devices such as indwelling catheters and pacemaker wires has been implicated in the rising incidence of SVC thrombus. Complete occlusion of the SVC is usually symptomatic and presents with a clinical picture similar to SVC syndrome. The importance of early detection and intervention was highlighted in this case, as the patient was initially asymptomatic after the onset of neurological symptoms. Treatment involved discontinuing heparin and starting the patient on Apixaban without a loading dose. This case study emphasizes the potential risk factors and complications associated with SVC thrombus and highlights the importance of early detection and intervention.

## Introduction

Superior vena cava (SVC) thrombus is an exceedingly rare but potentially life-threatening condition that occurs due to the formation of a blood clot in the superior vena cava. The superior vena cava is the vein that carries blood from the head, neck, and upper extremities to the heart. However, it has been found that the prevalence of SVC thrombosis is higher in certain populations, such as cancer patients, those with central venous catheters, and patients with SVC obstruction due to other causes [1]. Furthermore, the incidence of SVC thrombosis is also higher in patients with certain underlying medical conditions, such as malignancy, heart failure, and chronic obstructive pulmonary disease (COPD) [2]. In rare cases, the disease can also present itself without any underlying conditions. In this study, a case of a patient diagnosed with SVC thrombus will be discussed, as well as the management and treatment options implemented. The potential risk factors and complications associated with SVC thrombus will also be addressed, along with the importance of early detection and intervention.

## Case presentation

A 36-year-old African American female with a history of essential hypertension and Type 2 diabetes, along with anemia of chronic disease, obstructive sleep apnea, obesity, six days postpartum, and recently a history of preeclampsia, presented to the emergency room with sudden onset of confusion. The patient also experienced end-stage renal disease secondary to diabetic nephropathy with superimposed chronic tubulointerstitial nephritis and has been dialyzed on a Monday-Wednesday-Friday schedule since June 2021. Her husband reported that she was unable to move her right hand, had significant memory loss, and was not oriented to person and time. Her symptoms resolved upon presentation in the emergency room; however, the patient was admitted for further evaluation and treatment.

In July 2021, the patient was pregnant with twins, which led to acute, chronic renal failure and preeclampsia. Due to this renal failure, the patient was admitted for inpatient termination of pregnancy, and an IUD was placed to prevent further pregnancies. She was assessed by her primary care physician in May 2022 for a pap smear and confirmation of IUD placement.

In December 2022, the patient presented with abdominal pain in the emergency room, which led to the discovery that she was pregnant with twins at 25 weeks of gestational age. The patient was treated for preeclampsia at that time and was ultimately discharged four days after a scheduled c-section.

On the day of admission, she underwent computed tomography angiogram (CTA) head with/without contrast, which showed no hemorrhage or infarct. Moreover, her CT angiogram neck with/without contrast also came out to be negative, with no evidence of core infarction as assessed by CT Brain Perfusion. In addition, the CT Venogram head showed no evidence of dural venous sinus thrombosis. Diluted Russell Viper Venom Time (DRVVT) screen, Cardiolipin antibody (Ab), Glycoprotein B2 Ab, lupus anti coag, Factor V Leiden mutation, Factor II mutation, and methylenetetrahydrofolate reductase (MTHFR) mutation were all found to be negative. Antithrombin III activity was found to be 112% (normal: 83%-128%), activated partial thromboplastin time (aPTT) was 24.2 (normal: 24-36.5), protein S activity was 114% (normal: 64-149%), protein C activity was also 114% (normal: 70-140%), and anti-Xa was found to be 0.3 IU/mL (normal: 0.0-0.1 IU/mL). 

**Figure 1:**
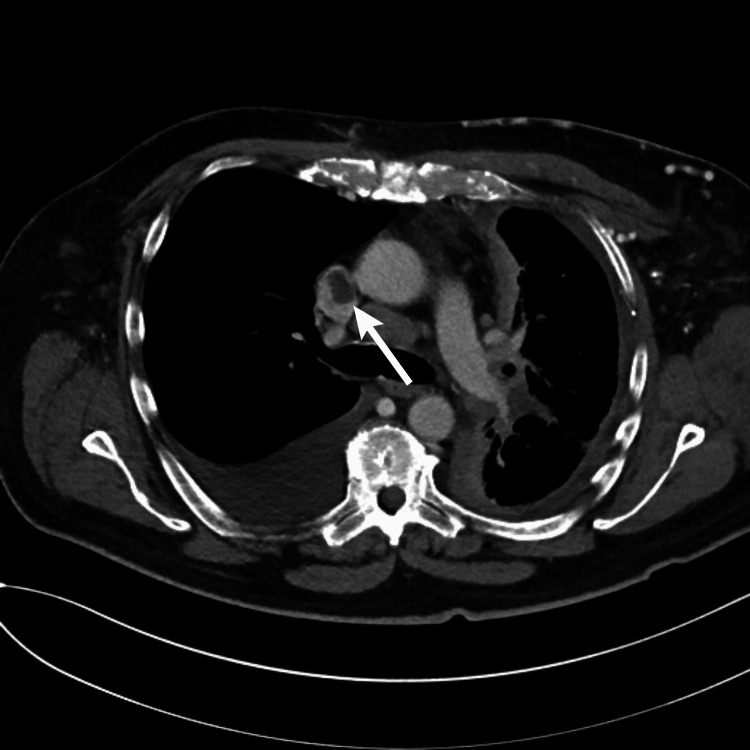
CT scan showing SVC thrombus marked with white arrow.

The following day, a magnetic resonance imaging (MRI) of the brain was performed, and an acute infarct in the left parietal lobe with no intracranial hemorrhage was noted. The patient was started on heparin. Furthermore, two days following her admission, transthoracic echocardiography was performed, which showed that the estimated ejection fraction was in the range of 60% to 65% with trivial pericardial effusion. However, the inferior vena cava was noted to be dilated, which was consistent with elevated central venous pressure. There was no evidence of hemodynamic compromise. Therefore, upper and lower extremity doppler were ordered to check for DVTs; however, no sonographic evidence of deep vein thrombosis was found. Five days after admission, a transesophageal echocardiogram was performed that depicted a large 1.3 x 1.4-centimeter echo density/mass. Upon correlation with CTA neck images, imaging was most consistent with SVC thrombus (Figure 1). The retrospective review of the CTA neck by the radiologist detected a rounded filling defect within the SVC, which supported a diagnosis of thrombus. The patient was cleared for discharge seven days after admittance. Heparin was discontinued, and Apixaban was started without a loading dose as outpatient treatment. Apixaban was planned to be taken for six months, and no surgical intervention was needed at the time. Further, aspirin was discontinued on recommendations from the neurology department.

## Discussion

Superior vena cava (SVC) thrombus, a rare but increasingly prevalent occurrence, is characterized by the formation of a thrombus within the lumen of the SVC. The increasing use of intravascular devices, such as indwelling hemodialysis catheters and pacemaker wires, has been implicated in the rising incidence of SVC thrombus [1]. Complete occlusion of the SVC is usually symptomatic and presents with a clinical picture similar to SVC syndrome, including but not limited to arm and facial edema, stridor, blurred vision, dyspnea, dizziness, positional headache, retroorbital pain, dysphagia, chest pain. The patient in this case report was initially asymptomatic following the onset of neurological symptoms, which delayed the diagnosis until proper imaging was utilized. Pregnancy, a known physiological hypercoagulable state, along with a past medical history of intra-dialytic hypotension which was reported by her nephrologist, and issues with catheter placement met all three criteria of Virchow's Triad: hypercoagulability, hemodynamic changes, and endothelial injury [3].

The SVC thrombus present in the patient went undiagnosed for five days following admission. The thrombus remained undetected despite utilizing multiple noninvasive imaging modalities, such as CT, ultrasound, transthoracic echocardiogram (TTE), and doppler. It was only through the utilization of a transesophageal echocardiogram that the diagnosis was finally made, with subsequent correlation to a previous CTA. In cases where the thrombus is uncomplicated, oral anticoagulation is the primary therapeutic option. However, surgical thrombectomy may be necessary for instances of large thrombi or hemodynamic instability [2,4].

The asymptomatic presentation of SVC thrombus poses a significant challenge for both its diagnosis and treatment. In such cases, clinicians must maintain a heightened level of suspicion to detect the thrombus before the patient's condition deteriorates. Failure to do so can result in delayed diagnosis and treatment, potentially leading to a more serious and unstable condition for the patient. Therefore, clinicians must be aware of the potential for asymptomatic SVC thrombus, as well as actively looking for signs of the condition during the examination through the use of imaging modalities such as CT and TEE. Lab tests, including CBC, D-dimer, and coagulation studies, should be used for the detection of life-threatening thrombi. This increased awareness and vigilance will aid in the early identification and management of SVC thrombus, ultimately improving outcomes for the affected patients. However, it is uncertain whether the parietal lobe infarct was caused by the SVC thrombus or another different process.

## Conclusions

This case study highlights the potential risks and complications associated with SVC thrombus, a rare but potentially life-threatening condition. Early detection and intervention are crucial to preventing serious complications, such as complete occlusion of the SVC. The patient's past medical history, including pregnancy, end-stage renal disease, and the use of indwelling catheters, were important risk factors that contributed to the development of an SVC thrombus. The use of proper imaging techniques was key to the eventual diagnosis of SVC thrombus, which allowed for the implementation of appropriate treatment measures. This case emphasizes the importance of considering SVC thrombus in patients with similar risk factors and presentations and the need for further research in this area to better understand the incidence, risk factors, and management of this condition.
